# Characterization of a β-glucanase produced by *Rhizopus microsporus *var. *microsporus*, and its potential for application in the brewing industry

**DOI:** 10.1186/1471-2091-7-23

**Published:** 2006-12-05

**Authors:** Klecius R Silveira Celestino, Ricardo B Cunha, Carlos R Felix

**Affiliations:** 1 Laboratório de Enzimologia, Departamento de Biologia Celular, Universidade de Brasília, Brasília, DF, CEP 70910-900, Brazil; 2 Centro Brasileiro de Serviços e Pesquisas em Proteínas (LB QP)-Divisão de Química Analítica, Instituto de Química, Universidade de Brasília, Brasília, DF, CEP 70910-900, Brazil

## Abstract

**Background:**

In the barley malting process, partial hydrolysis of β-glucans begins with seed germination. However, the endogenous 1,3-1,4-β-glucanases are heat inactivated, and the remaining high molecular weight β-glucans may cause severe problems such as increased brewer mash viscosity and turbidity. Increased viscosity impairs pumping and filtration, resulting in lower efficiency, reduced yields of extracts, and lower filtration rates, as well as the appearance of gelatinous precipitates in the finished beer. Therefore, the use of exogenous β-glucanases to reduce the β-glucans already present in the malt barley is highly desirable.

**Results:**

The zygomycete microfungus *Rhizopus microsporus var. microsporus *secreted substantial amounts of β-glucanase in liquid culture medium containing 0.5% chitin. An active protein was isolated by gel filtration and ion exchange chromatographies of the β-glucanase activity-containing culture supernatant. This isolated protein hydrolyzed 1,3-1,4-β-glucan (barley β-glucan), but showed only residual activity against 1,3-β-glucan (laminarin), or no activity at all against 1,4-β-glucan (cellulose), indicating that the *R. microsporus var. microsporus *enzyme is a member of the EC 3.2.1.73 category. The purified protein had a molecular mass of 33.7 kDa, as determined by mass spectrometry. The optimal pH and temperature for hydrolysis of 1,3-1,4-β-glucan were in the ranges of 4–5, and 50–60°C, respectively. The Km and Vmax values for hydrolysis of β-glucan at pH 5.0 and 50°C were 22.39 mg.mL^-1 ^and 16.46 mg.min^-1^, respectively. The purified enzyme was highly sensitive to Cu^+2^, but showed less or no sensitivity to other divalent ions, and was able to reduce both the viscosity and the filtration time of a sample of brewer mash. In comparison to the values determined for the mash treated with two commercial glucanases, the relative viscosity value for the mash treated with the 1,3-1,4-β-glucanase produced by *R. microsporus var. microsporus*. was determined to be consistently lower.

**Conclusion:**

The zygomycete microfungus *R. microsporus var. microsporus *produced a 1,3-1,4-β-D-glucan 4-glucanhydrolase (EC 3.2.1.73) which is able to hydrolyze β-D-glucan that contains both the 1,3- and 1,4-bonds (barley β-glucans). Its molecular mass was 33.7 kDa. Maximum activity was detected at pH values in the range of 4–5, and temperatures in the range of 50–60°C. The enzyme was able to reduce both the viscosity of the brewer mash and the filtration time, indicating its potential value for the brewing industry.

## Background

1,3-1,4-β-Glucans are polysaccharides, components of the cell walls of higher members of the *Poaceae *family. They areparticularly abundant in the endosperm cell walls of commercially valuable cereals such as barley, rye, sorghum, oats and wheat [1]. Structurally, these polysaccharides are linear glucans of up to 1,200 β-D-glucosyl residues linked through β-1,3 and β-1,4 glycosyl bonds. Variations in the proportions of β-1,3-(25–30%) and β-1,4-linkages, and in the length of the mixed-linked segments are currrently reported [2]. During malt production, partial hydrolysis of barley β-glucans begins with seed germination [13]. However, the endogenous 1,3-1,4-β-glucanases are heat inactivated, and the remaining high molecular weight β-glucans may cause severe problems such as increased brewer mash viscosity and turbidity[14]. Increased viscosity impairs pumping and filtration, causing lower efficiency, reduced yields of extracts, and lower filtration rates, as well as the appearance of gelatinous precipitates in the finished beer [2]. Thus, both the level of glucan-hydrolysing activities achieved during germination and the amounts of their substrates, mainly 1,3-1,4-β-glucan, are important factors in the production of high quality malts. Addition of exogenous 1,3-1,4-β-glucanases to the mash could therefore be an outstanding option for improving the brewing process. However, the β-glucanases currently marketed do not really meet the brewing industry's needs, mainly due to economic factors. Novel 1,3-1,4-β-glucanases with uncommon features would be highly desirable. Here we report on the production, purification and partial characterization of a 1,3-1,4-β-glucanase produced by *R. microsporus var. microsporus*, considering as well its potential for use in the brewing industry.

## Results and Discussion

### Enzyme production

**Figure 1:**
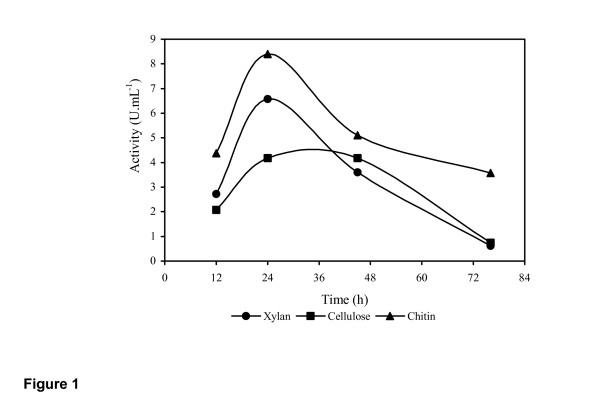
Time course of production of 1,3-1,4-β-glucanase by *Rhizopus microsporus var. microsporus *in the presence of 0.5% of either xylan, cellulose or chitin at a temperature of 40°C and at 120 rpm

A 1,3-1,4-β-glucan-degrading filamentous fungus was isolated from a malt silo in a brewery. This zygomycete microfungus was identified as *Rhizopus microsporus var. microsporus *by rcDNA analysis. It grew strongly in liquid medium containing chitin as the sole carbon source, and produced substantial amounts of β-glucanase activity (Figure 1) which was able to fully hydrolyze barley β-glucan (1,3-1,4-β-glucan). The specificity of substrate hydrolysis by this enzyme (Table 2) fully supports the assumption that it belongs to the 3.2.1.73 category. The inducible nature of 1,3-1,4-β-glucanase production has already been reported for 1,3-β-glucanase from *Trichoderma sp*. [27]. Although cellulose and xylan were also inducers, the levels of enzyme secreted in the presence of these carbohydrates were considered smaller than the activity induced by chitin. In cultures grown under agitation (120 rpm) at 40°C, the enzyme activity increased from a minimum to a maximum level within 24h of growth. It has been reported that several other microrganisms, including *Bacillus sp*. [15]*Trichoderma sp*. [16], *Talaromyces emersonii *[17], and *Rhizobium sp*. [2], produce 1,3-1,4-β-glucanase enzymes which are currently used in the brewing industry. However, depending on the substrate (β-glucan) used as inducer, production of the glucanases for industrial application may be very costly, to the point of being considered economically prohibitive [28]. Chitin, on the other hand, is a relatively cheap and readily available carbon source in comparison to barley β-glucan and laminarin. The ability, therefore, of *R. microsporus var. microsporus *to produce 1,3-1,4-β-glucanase in the presence of chitin, favors its use on an industrial scale.

**Table 2:** Hydrolysis of glucan substrates by the *R. microsporus *purified β-glucanase. ΔAbs _550 nm _represents the net absorbance of the reaction mixture after incubation for 0.5 h with the enzyme at 50°C.

Substrate	**ΔAbs **_550 nm_
Laminarin (1,3-β-glucan)	0.029
Chitin	0.001
CM-cellulose (1,4-β-glucan)	0.001
Xylan	0.026
Manan	0.000
Barley β-glucan (1,3-1,4-β-glucan)	0.826

### Enzyme Purification

**Figure 2:**
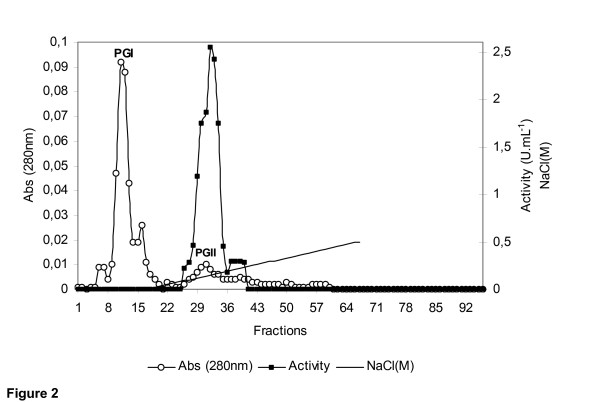
Ion exchange (SP-Sepharose column) chromatography of the concentrated culture filtrate of *Rhizopus microsporus var. microsporus *grown in liquid medium containing 0.5% chitin.

**Figure 3:**
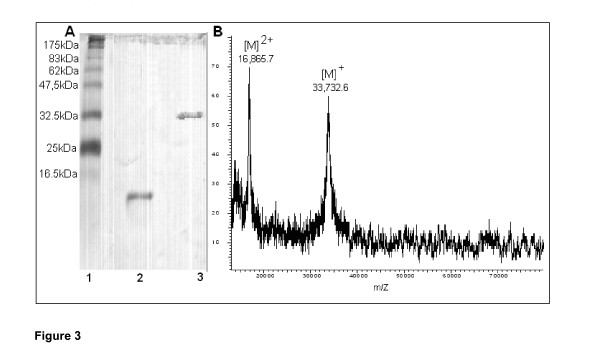
SDS-PAGE (A) and MALDI-TOF mass spectrometry (B) analysis of the purified 1,3-1,4-β-glucanase from *Rhizopus microsporus var. microsporus*. A: line 1, molecular weight markers; line 2, PGI protein fraction; line 3, PGII protein fraction.

The culture supernatant of *R. microsporus var. microsporus *grown in liquid medium containing chitin was concentrated 10-fold by ultrafiltration, using a 10 kDa cut-off membrane. No 1,3-1,4-β-glucanase activity was found in the filtrate. Chromatography of the concentrate on a Sephacryl S-100 gel filtration column (not shown) followed by chromatography on an SP-Sepharose ion exchange column resulted in elution of two peaks of proteins (PGI and PGII) (Figure 2). While the PGI proteins were inactive, the PGII protein (fractions 22–34) showed substantial activity against 1,3-1,4-β-glucan. A summary of the purification steps of the 1,3-1,4-β-glucanase produced by the *R. microsporus var. microsporus *is shown in table 1. The enzyme was purified (Figure 3) 55.529-fold with a yield of 114.912% and a specific activity of 12.596 U.mg^-1^. The molecular mass of the PGII protein was 36.5 kDa, as indicated by SDS-PAGE analysis (Figure 3). This value is comparable to that (33.7 kDa) determined by mass spectrometry analysis for this enzyme (Figure 3). While 1,3-1,4-β-glucanases from *Bacillus sp*. have smaller molecular masses varying in the range of 25–30 kDa [2], the enzymes from *Clostridium thermocellum *(38 kDa) [34], *Bacteroides succinogenes *(37 kDa) [33] and *Talaromyces emersonii *(40.7 kDa) [2] showed comparable molecular mass values.

**Table 1:** Summary of the purification protocol of the 1,3-1,4-β-glucanase produced by *Rhizopus microsporus var. microsporus*.

Steps	**Total Protein(mg)**	**Total Activity(U)**	**Specific activity(U.mg**^-1^**)**	**Purification (-fold)**	**Yield(%)**
Concentrated crude extract	1.574	0.228	0.197	1	100.000
Sephacryl S-100 eluate	0.235	0.227	0.966	4.915	99.561
SP – Sepharose eluate	0.021	0.262	12.596	55.529	114.912

### Enzyme specificity

The *R. microsporus *purified β-glucanase was tested for its ability to hydrolyze several other glucan substrates. As may be seen in table 2, only the barley β-glucan was efficiently hydrolyzed, as indicated by the much higher net absorbance. In comparison to the activity against the 1,3-1,4-β-glucan, very low or no activity at all was shown by the enzyme against the substrates laminarin (1,3-β-glucan) and CM-cellulose (soluble 1,4-β-glucan), indicating clearly that the enzyme may be taken as a member of the EC 3.2.1.73 enzyme category.

### Effect of pH and temperature optima

**Figure 4:**
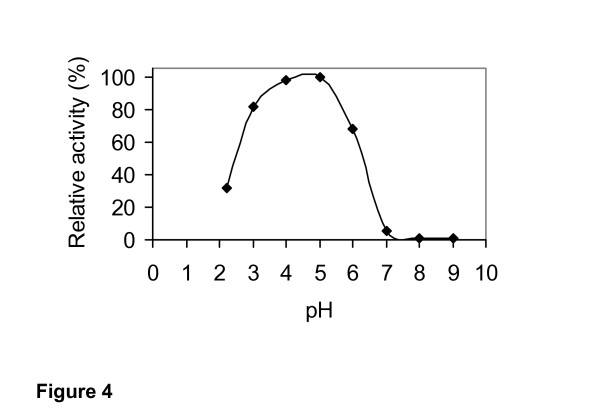
Effect of pH on the activity of the purified 1,3-1,4-β-glucanase from *Rhizopus microsporus var. microsporus*, at 50°C.

**Figure 5:**
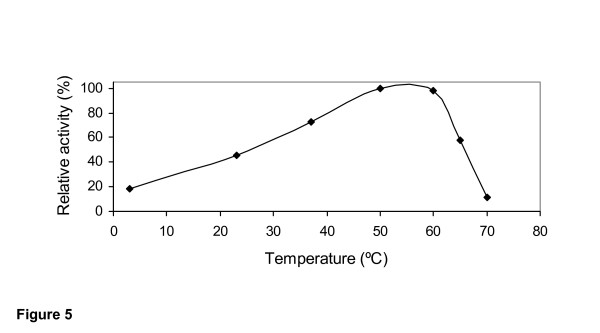
Effect of temperature on the activity of the purified 1,3-1,4-β-glucanase from *Rhizopus microsporus var. microsporus*, at pH 5.0.

The effect of pH and temperature on the activity of the purified 1,3-1,4-β-glucanase from *Rhizopus microsporus var. microsporus *is shown in figures 4 and 5, respectively. At 50°C, the enzyme showed substantial activity in the pH range of from 2 to 6. Maximal activity was recorded in the range of from 4 to 5. No enzyme activity was detected at pH higher than 6 (Figure 4). At pH 5.0, the purified enzyme was substantially active in the temperature range from 20°C to 65°C. Maximal activity was detected at 50°C and 60°C, indicating that the optimal temperature for glucan hydrolysis is 55°C (Figure 5). The optima pH and temperature values determined for the purified 1,3-1,4-β-glucanase from *R. microsporus var. microsporus *were similar to those determined for 1,3-1,4-β-glucanases from several other fungi and bacteria [2]. In addition, these values are comparable to those presented by enzymes currently being used in the brewing industry [11,2]. The purified 1,3-1,4-β-glucanase retained 100% and 87% of its activity after incubation for 2 h and 24 h, respectively, at 50°C. The half-lives of the enzyme at the temperatures of 60°C and 70°C were found to be 10 min and 1 min, respectively. At 50°C, the half-life was 72 h (data not shown). For hydrolysis of β-glucan by a novel 1,3-1,4-β-glucanase produced by *Bacillus halodurans C-125*, the pH optimum was between 6 and 8, and the temperature optimum was 60°C. After 2 h incubation at 50°C and 60°C, the residual activity remained 100% and 50%, respectively. The enzymatic activity was abolished after 3 min incubation at 70°C. The optimum temperature for hydrolysis of lichenan by a 1,3-1,4-β-glucanase from *Bacteroides succinogenes *at ph 6.0 was 50°C [33].

### Effect of metal ions

The effect of several ions on the activity of the purified 1,3-1,4-β-glucanase produced by *R. microsporus var. microsporus *is shown in Table 3. The enzyme was sensitive to copper and fairly sensitive to zinc and manganese, but insensitive to magnesium, calcium and aluminum (Table 3). Glucanases produced by *Rhizopus oryzae *[29], *Bacillus clausii *[30], *Bacillus halodurans *[32] and *Trichoderma harzianum *[31] show similar sensitivity to the divalent metal ion copper.

**Table 3:** Effect of metal ions on the activity of the purified 1,3-1,4-β-glucanase from *Rhizopus microsporus var. microsporus*.

**Ion **	**Residual activity (%)**
Control	100
Cu^+2 ^(12 mM)	0.3
Mg^+2 ^(12 mM)	95.2
Fe^+3 ^(12 mM)	89.6
Zn^+2 ^(12 mM)	65.0
Mn^+2 ^(12 mM)	62.3
Ca^+2 ^(12 mM)	105.9
Al^+3 ^(12 mM)	109.8

### Kinetic Parameters

**Figure 7:**
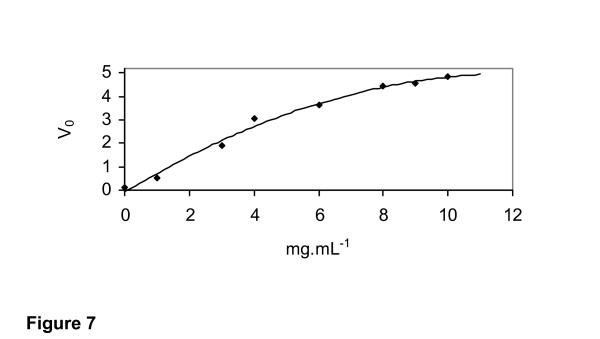
Hydrolysis (μmol·min^-1^·mL^-1^) of β-glucan by the purified 1,3-1,4-β-glucanase from *Rhizopus microsporus var. microsporus*, in the presence of different concentrations of 1,3-1,4-β-glucan.

The purified 1,3-1,4-β-glucanase produced by *R. microsporus var. microsporus *hydrolyzed 1,3 – 1,4-β-glucan in a Michaelis-Menten fashion (Figure 7). Kinetic parameters were calculated using a Michaelis-Menten plot with a non-linear regression data analysis program [10]. Values of 19.8 mg.mL^-1^, 12.7s^-1 ^and 16.5 U.mL^-1 ^were determined for Km, Kcat and Vmax, respectively. Km values of 1.2 – 1.5 mg.mL^-1 ^for hydrolysis of barley β-glucan and 0.8 – 2 mg.mL^-1 ^for lichenan were reported for the 1,3-1,4-β-glucanase produced by Bacillus sp [2]. Values of 1,296 ± 51, 2.50 ± 0.09, and 518 were reported for Kcat (s^-1^), Km (mg.mL^-1^) and Kcat/Km (s^-1^.M^-1^) respectively, for hydrolysis of lichenan by a 1,3-1,4-β-glucanase produced by *Bacteroides succinogenes*. [33].

### Capillary Viscosimetry and Filtration rate

The specific filtration rate and specific viscosity rate of the mash after incubation with the 1,3-1,4-β-glucanase from *R. microsporus var microsporus *were compared with those values calculated for two commercial β-glucanases currently used in the brewing industry. The results are shown in Tables 4 and 5. Even at lower enzyme concentration, the 1,3-1,4-β-glucanase from *R. microsporus var microsporus *caused a higher reduction in the filtration rate (20.4%) of the mash (table 4). Similar results were obtained for the specific viscosity of the brewer's mash after treatment with the three β-glucanases (table 5).

**Table 4:** Total protein in the enzyme samples, filtration time, filtration time reduction and specific filtration time reduction of the brewer's mash not treated or treated with enzymes.

	**Total Protein in the enzyme sample (μg)**	**Filtration time (seconds)**	**Filtration time reduction (%)**	**Specific filtration time reduction (%/μg)**
Control	-	274	-	-
*R. microsporus var microsporus *enzyme	11.0	218	20.4	1854.5 × 10^-3^
Commercial enzyme A	394	195	29.9	75.9 × 10^-3^
Commercial enzyme B	373	176	35.8	95.8 × 10^-3^

**Table 5:** Total protein in the enzyme samples, viscosity, viscosity reduction and specific viscosity reduction of the brewer's mash not treated or treated with enzymes.

	**Total Protein in the enzyme sample (μg)**	**Viscosity (cP)**	**Viscosity reduction (%)**	**Specific viscosity reduction (%/μg)**
Control	-	1.070	-	-
*R. microsporus var microsporus *enzyme	110	1.020	4.7	763 × 10^-3^
Commercial enzyme A	394	1.002	6.3	14.2 × 10^-3^
Commercial enzyme B	373	1.001	6.4	17.3 × 10^-3^

## Conclusion

The zygomycete *Rhizopus microsporus var. microsporus *produced a 1,3-1,4-β-D-glucan 4-glucanhydrolase (EC 3.2.1.73) which could hydrolyze β-D-glucan substrate containing both 1,3- and 1,4-bonds. Its molecular mass as determined by both electrophoresis and mass spectrometry (MALDI-TOF) was about 33.7 kDa. Its optimum pH and temperature were found to be in the ranges of 4–5 and 50–60°C, respectively. Kinetic analysis and its capacity to reduce both the viscosity of the brewer mash and the filtration time, indicate the possibility to use this enzyme in the brewing industry.

## Methods

### Chemicals

Barley 1,3-1,4-β-glucan, chitin, CM-cellulose, manan, xylan, laminarin, molecular mass standard proteins and sodium dodecyl sulfate (SDS) were from Sigma Chemical Co., USA. Sephacryl S-100 and SP-Sepharose were from Pharmacia-LKT, Sweden. All other chemicals were of analytical grade.

### Organism and enzyme production

The aerobic zygomycete microfungus *Rhizopus microsporus var. microsporus *was isolated from a malt silo. The fungus was maintained at 4°C, after growing for 48 hours in TLE modified solid medium [(0.5% chitin, 0.2% KH_2_PO_4_, 0.14%(NH_4_)_2_SO_4_, 0.03% MgSO_4_.7H_2_O, 0.0152% CaCl_2_, 0.02% glucose, 1.0 mL of 0.01% trace elements solutions (Fe^+2^, Mn^+2^, Zn^+2^, Co^+2^), 0.003% bactopeptone, 0.003% urea, and 2% agar, pH 6.8)], at 40°C.

For enzyme production, one liter Erlemeyer flasks containing 250 mL of the liquid medium (TLE with no agar), were inoculated with 150 cm^2 ^blocks of solid medium taken from 2-day old *R. microsporus var. microsporus *cultures. Liquid cultures were then incubated for 24 hours with agitation (120 rpm) at 40°C. The culture supernatants were then separated from the mycelium by filtration, using filter paper. The supernatants were then freeze-dried and used either for enzyme assay or enzyme purification as described in the following sections.

### Enzyme assay

1,3-1,4-β-glucanase activity was assayed by the reducing-sugar method [6] with β-1,3-β-1,4-glucan as the substrate. The assay system consisted of 50 μL of 1% (wt/vol) β-glucan dissolved in 100 mM sodium acetate buffer, pH 5.0, and 50 μL enzyme sample. The reaction was allowed to proceed for 30 min at 50°C, and was then stopped by the addition of 300 μL dinitrosalicylate reagent [6], and 5 min of boiling. The absorbance of the reaction mixture was determined at 550 nm using a Perkin Elmer mod. Lambda 11/Bio spectrophotometer. The amount of reducing sugar produced was determined using a curve constructed with glucose as standard. One unit of enzyme was defined as the amount of protein necessary to produce one μmol of reducing sugars.min^-1^.

The assays for xylanase, cellulase, 1,3-β-glucanase and mananase were performed as for 1,3-1,4-β-glucanase, except for the use of the substrates carboximetilcellulose, laminarin and manan, respectively. For chitinase the enzyme system consisted of 100 μL of enzyme sample, regenerated chitin 0,5% in 50 mM sodium acetate buffer, pH 5.2 [35]. The reaction was allowed to run for 12h at 37°C and stopped by addition of dinitrosalycilic reagent. The amount of reducing sugar produced was quantified using a standard curve constructed with glucose.

### Purifications of the 1,3-1,4-β-glucanase from *Rhizopus microsporus var. microsporus*

The supernatants from cultures of *R. microsporus var. microsporus *grown in liquid medium containing β-glucan were concentrated by ultrafiltration [(Amicon system; 10 k-Da cut-off membrane (PM10)]. Aliquots of concentrated β-glucanase were loaded on a Sephacryl S-100 gel column (2.5 × 40 cm), equilibrated and eluted with 50 mM sodium acetate buffer, pH 5.0. Elution was performed at a flow rate of 24 mL.h^-1^, and fractions of 4.0 mL were collected. Active fractions were pooled and applied on a SP-Sepharose ion-exchange column (3.0 × 15 cm), previously equilibrated and eluted with 50 mM sodium acetate buffer, pH 5.0, and further eluted with a linear gradient formed with 100 mL of the acetate buffer and 100 mL of the same buffer containing 1 M NaCl. Elution was carried out at a flow rate of 24 mL.h^-1^, and fractions of 4 mL were collected. The resulting active fractions were pooled and dialyzed overnight against distilled water at 4°C, concentrated by ultrafiltration as above, and stored at -20°C until their use.

### Protein determination

Protein was determined by the Bradford method [7], with bovine serum albumin as standard.

### Electrophoresis

Enzyme samples were examined by electrophoresis under denaturing conditions in polyacrylamide slab gels (SDS-PAGE) as described by Laemmli [8]. Protein bands in the gel were visualized by the silver staining method [9].

### Mass spectrometry

1,3-1,4-β-glucanase was analyzed by matrix-assisted laser desorption ionization-time of flight (MALDI-TOF) mass spectrometry with a Reflex IV mass spectrometer (Bruker Daltonik, Bremen, Germany) in linear positive mode. The purified enzyme sample (50 μg) was dissolved in 50 μL of 0.1 % (v/v) TFA, from which 1 μL was mixed with 1 μL of a saturated matrix solution of sinapinic acid dissolved in 50% (v/v) acetonitrile and 0.1% (v/v) trifluoroacetic acid, and applied to the MALDI plate. BSA was used for external mass calibration.

### Effects of ions

The effects of several metallic ions (Cu^+2^, Mg^+2^, Fe^+3^, Zn^+2^, Ca^+2^, and Al^+3^) on the purified 1,3-1,4-β-glucanase were tested measuring the activity of the enzyme at 50°C (see 1,3-1,4–β-glucanase assay) in the presence of the ions.

### pH and temperature optima

The effect of temperature on the enzyme was carried out at the temperature range of from 4° to 70°C, at pH 5.0 (in 50 mM sodium acetate buffer). The optimum pH value was determined by monitoring the enzyme activity at 50°C at pH values from 3.0 to 9.0. The following buffers were used: pH 3.0 – 6.0, 50 mM sodium acetate; pH 7.0, 50 mM sodium phosphate; and pH 8.0 – 9.0, 50 mM tris-HCl.

### Kinetic Parameters

For determination of kinetic parameters, the enzyme assays were performed at 50°C, using 1,3-1,4-β-glucan at concentrations varying from 0.05 to 2.0 % dissolved in 50 mM sodium acetate buffer, pH 5.0. Km, Kcat and Vmax values were obtained using a Michaelis-Menten plot with a non-linear regression data analysis program [10].

### Preparation of the mash

12.5 g of malt was triturated in a hammer mill (MAROTEC), drizzled into a sieve of 0.2 mm spacing, and dissolved in 50 mL of sodium acetate buffer (100 mM, pH 5.5), pre-heated to 45°C. Reaction started with 1.0 mL of enzyme sample taken from chromatography on a Sephacryl S-100 column, and allowed to proceed for 30 min at 45°C, followed by other periods of 10 min at 50°C, 15 min at 60°C, 60 min at 70°C, and 5 min of boiling. The reaction was then stopped by the addition of 100 mL of cold water and immediate cooling in an ice-water bath at about 20°C.

### Capillary viscosimetry

Decrease in mash viscosity was measured by capillary viscosimetry using an Oswald viscosimeter [12,20]. Samples of 30 mL of mash were filtered using filter paper and placed in a viscosimeter at 20°C. Mash viscosity in the absence of enzyme was used as a control. The specific viscosity rate was calculated using the following equations:

μ_mash _= (μ_water _× T_mash _× ρ_mash_)/(T_water _× ρ_water_)     (1)

Δμ = (μ_mash control _– μ_mash_) × 100/(μ_mash control_)     (2)

Δμφ = Δμ/δ     (3)

Where μ is the viscosity, T is the flow time, Δμ is viscosity reduction, δ is total protein, Δμφ is specific viscosity rate and ρ is the density.

### Filtration rate

The filtration rate was determined by filtration of 50 mL of mash through a filter paper [12]. Filtration rate in the absence of enzyme was used as a control. The specific filtration rate was calculated using the following equations:

Δ_ψ _= (ψ_mash control _– ψ_mash_) × 100/(ψ_mash control_)     (4)

Δ_ψφ _= Δψ/δ     (5)

Where ψ is the flow time, Δψ is the filtration time of 50 mL, δ is total protein and Δψφ is the specific filtration reduction.

## Authors' contributions

KRSC conceived of the study and participated in its design, performed all experiments, data quantification and was involved in the literature search and data interpretation. RBC participated and supported the mass spectrometry analysis with a Reflex IV mass spectrometer. CRF participated in the design and coordination of the study, as well as on its supervision, and helped to draft the manuscript. All authors read and approved the final manuscript.

**Figure 6:**
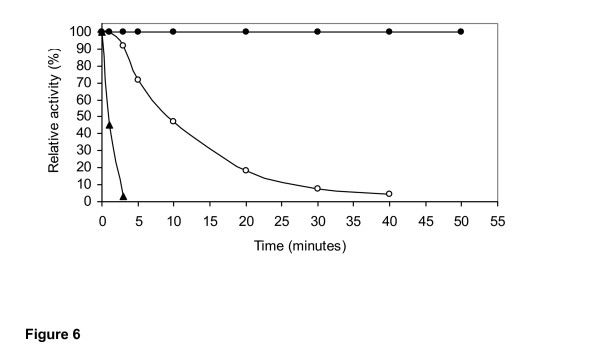
Thermostability of the purified 1,3-1,4-β-glucanase from *Rhizopus microsporus var. microsporus*, at temperatures of 50°C (●), 60°C (○) and 70°C (▲), at pH 5.0.
